# Combined Hand Gesture — Speech Model for Human Action Recognition

**DOI:** 10.3390/s131217098

**Published:** 2013-12-12

**Authors:** Sheng-Tzong Cheng, Chih-Wei Hsu, Jian-Pan Li

**Affiliations:** Department of Computer Science and Information Engineering, National Cheng Kung University, No.1, University Road, Tainan City 701, Taiwan; E-Mails: stcheng@mail.ncku.edu.tw (S.-T.C.); keyboard802@hotmail.com (J.-P.L.)

**Keywords:** hand gesture detection, hand gesture recognition, speech recognition, human behavior

## Abstract

This study proposes a dynamic hand gesture detection technology to effectively detect dynamic hand gesture areas, and a hand gesture recognition technology to improve the dynamic hand gesture recognition rate. Meanwhile, the corresponding relationship between state sequences in hand gesture and speech models is considered by integrating speech recognition technology with a multimodal model, thus improving the accuracy of human behavior recognition. The experimental results proved that the proposed method can effectively improve human behavior recognition accuracy and the feasibility of system applications. Experimental results verified that the multimodal gesture-speech model provided superior accuracy when compared to the single modal versions.

## 1. Introduction

Hand gestures are one of the primal communication methods. Even in environments where language communication cannot be made, hand gestures can deliver messages to achieve the purpose of communication. In a digital family care system, hand gestures are the best way for people with language disorders, or those who are mobility handicapped, to express themselves. As more studies have focused on human-computer interaction [1–3], how to use automatic hand gesture detection and recognition systems in natural environments [4] has attracted wide research attention.

**Figure 1.**
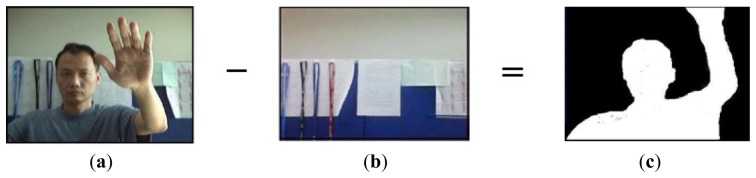
Moving object detection. (**a**) Front image; (**b**) Background image; (**c**) Moving object.

In a hand gesture recognition system [5–11], hand gesture detection technology is critical to the accuracy of hand gesture recognition. Thus, how to accurately detect hand gesture areas in image sequencing of natural environments is a topical subject. Traditionally, hand gesture detection is based on skin color [12,13], and detects hand gesture areas with statistics of color, space, and predefined conditions. However, if hand gestures are detected only by skin color, the complicated backgrounds or light source variations in natural environments may cause undesired detection effects in human-computer interaction. On the other hand, from the perspective of object detection, background display has little variation. Thus, traditional movement object detection technology uses a prior background model [14,15]. When an object enters into a prior background model, moving object areas in the image can be extracted by detecting the differences between images and background model, as shown in Figure 1. In [15], the author detects the moving object by subtracting the background model, and then tracks the moving object using a Kalman filter. The author builds a database of contour features for moving objects, and the occluded region is restored by searching for the similar moving objects in the database.

This study proposes a method for dynamic hand gesture detection, which combines motion estimation and skin color detection technology, and uses horizontal and vertical projection of binary images to detect the dynamic hand gesture areas. Without prior training, dynamic hand gesture areas can be effectively detected in complicated background environments.

In a human behavior recognition system, the traditional method describes human behavior through predefined features; however, this method lacks robustness due to feature variations. For example, the features of speed and body size changes are different when running and walking. If the features of speed and body size changes are used to describe human behaviors, and a threshold value is defined for recognition, the recognition effect may be inaccurate and lacks robustness. Thus, subsequent studies have proposed the statistic model concept to describe the variations of each attribute in order to increase the recognition rate of human behaviors.

As discussed above, robustness has a significant impact on human behavior recognition. For the dynamic recognition system, this study proposes a Kalman Filter-based dynamic gesture feature estimation, which considers relations between adjacent gesture areas based on time, and obtains robust features to describe hand gestures. Furthermore, repeated gesture segments in image sequencing are detected through an autocorrelation function operation in order to improve the variations of feature extraction, as caused by repeated hand gestures, and the dynamic hand gesture recognition rate in natural environments.

Speech is a natural and direct method of human expression. In a family care system, speech is a medium for direct expression of demands for the individuals with disabilities. Thus, this study further considers speech information in human behavior recognition. First, the input speech sequence is extracted through a microphone, and speech feature parameters are obtained by extracting Mel-scale Frequency Cepstral Coefficients. Furthermore, repeated speech segments are detected through autocorrelation function operations to reduce variations in feature extraction. Finally, the recognition rate of human behaviors can be improved by integrating the technology of a multimodal model of gestures with a speech model.

Human-computer interaction can be achieved by extraction and recognition of human behaviors. This study develops a dynamic hand detection and recognition system, which can be used for human-computer behavior recognition. Finally, a speech recognition system is incorporated and the human behavior recognition rate is improved using the multimodal model technique. The rest of this paper is organized as follows: Section 2 describes research and pertinent methods related to ours. Section 3 presents dynamic hand gesture recognition technology. Section 4 presents speech recognition technology. Section 5 presents multimodal model integration. The experimental results are presented in Section 6. Finally, Section 7 concludes the presentation of the proposed dynamic hand gesture detection and recognition system.

## 2. Related Work

In previous hand gesture detection research, skin color detection is often used in hand gesture detection methods [16]. Hand gesture areas can be detected through skin color statistics in RGB color space and predefined conditions. In [17] a support vector machine (SVM) was used to cut skin color. As SVM must first select samples for training, and classification results depend on sample accuracy, it is not suitable for complicated background environments. In response to this problem, hand gesture segmentation technology uses color skin detection, and acquires the threshold value of the color space using a statistical method. The hand gesture area can thus be obtained after detection of the input image. However, this human-machine interface method is restricted in application due to complicated background factors, facial skin color, and rays of light.

In behavior recognition research, the authors of [18] proposed a new feature representation algorithm of motion field sequence, and used the projection of sports features in time and space to compare with action samples recorded before an event, in order to further recognize human behavior or intention. This method considers motion features in an image as basis for recognition of human behaviors. If several objects move in the images, the recognition effect obviously declines due to extraction of excessive motion feature variations. This method is not suitable for complicated backgrounds, and is thus restricted in human-machine interface applications. Many studies of human behavior recognition consider the extraction of robustness parameters. The authors of [19] used a Gaussian Mixture Model to describe the variation of each feature attribute in order to increase the human behavior recognition rate. In [20], the authors proposed facial expression recognition system using multi-class AdaBoost with dynamic time warping, or by using support vector machine on the boosted feature vectors. The identification of hand motions becomes more difficult as the number of hand motion types increases [21]; the work described in [5] includes detecting and tracking bare hand in cluttered background using skin detection and hand posture contour comparison algorithm after face subtraction, recognizing hand gestures. Hand gesture recognition for real-life applications is very challenging because of its requirements on the robustness, accuracy and efficiency [6]. In [11], the authors described a nonspecific person gesture recognition system, which consists of sensor data collection, segmentation and recognition.

RGB-D cameras are novel sensing systems that capture RGB images along with per-pixel depth information [22]. In [6] a hand gesture recognition system that is robust to cluttered backgrounds, because the hand shape is segmented from the background using a depth camera is proposed. In [23] a Kinect-style depth camera is used for building dense 3D maps of indoor environments. They proposed a full 3D mapping system that utilizes a novel joint optimization algorithm combining visual features and shape-based alignments. In [24] a new human-gesture captures sensor solution to use natural human body language for human-virtual human interaction. In [25] a new visual representation for hand motions based on the motion divergence fields, which can be normalized to gray-scale images.

## 3. Dynamic Hand Gesture Recognition Technology

**Figure 2.**
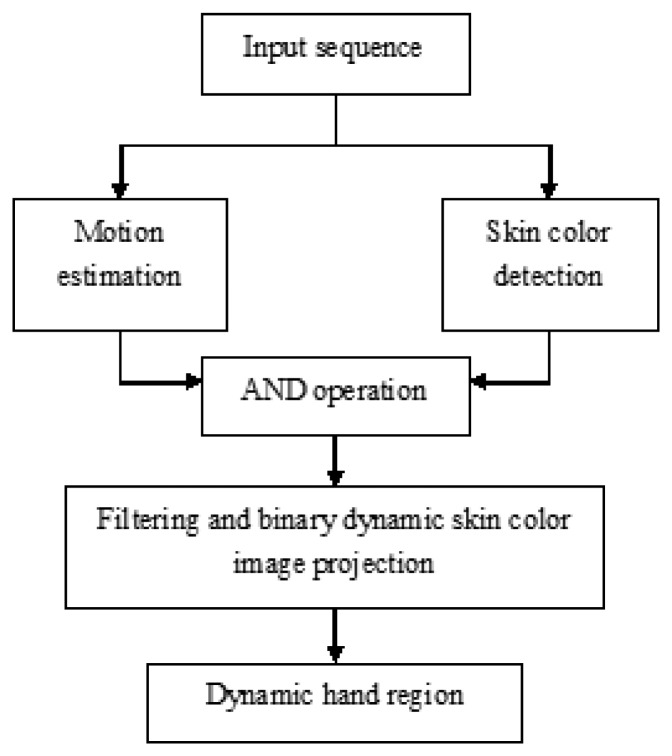
Dynamic hand gesture detection flow chart.

Hand gesture detection is different from the features of traditional skin color. This study proposes a dynamic gesture recognition algorithm using combined motion and skin color cues. The dynamic gesture region is determined by vertical and horizontal vector testing [26]. Based on this method, this paper uses a self-function to extract the eigenvector model. Without prior training, it can effectively detect dynamic hand gesture areas in complicated background environments. Figure 2 shows the systemic flow chart of the dynamic hand gesture detection method, which is divided into four parts: motion estimation, skin color detection, AND operation, and binary image projection.

The dynamic hand gesture detection flow chart is described, as follows: first, a series of input image sequences are extracted through a webcam. The dynamic object can be obtained through motion estimation, and skin color area is extracted from the images through skin color detection. Dynamic skin color images can be obtained through interactive operations. Finally, dynamic hand gesture areas can be obtained by projecting dynamic skin color in the bindery images.

### 3.1. Motion Estimation

**Figure 3.**
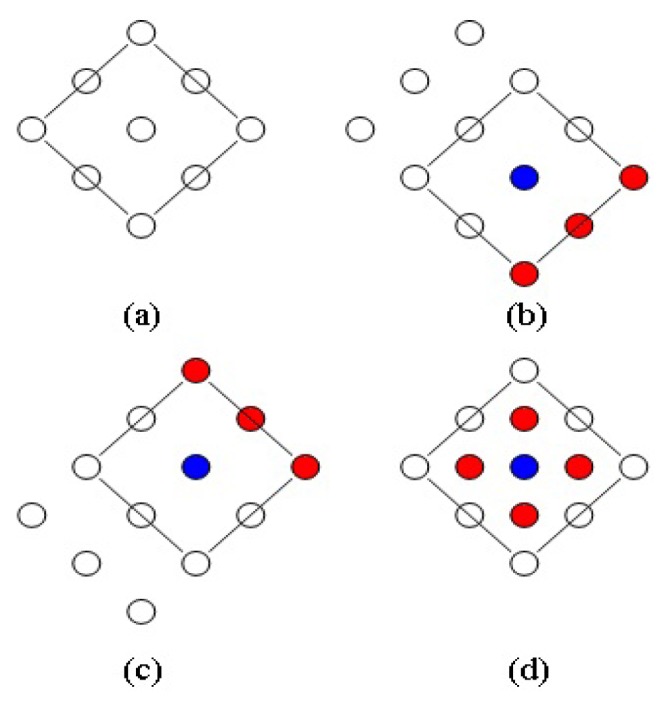
Diamond search method.

In order to rapidly detect moving objects in an image, this study applies the diamond search algorithm [27], as shown in Figure 3. It is based on search patterns, and uses a block match algorithm to detect moving objects in images.

In Figure 3a, diamond sample plates are used during the search. If the majority of matched blocks are located on the blue point, as seen in Figure 3b, the blue point is used as the center point, and another diamond sample plate is opened. Thus, new diamond search plates can be obtained after three red points are added in the right lower direction of the blue point. It is assumed that, the majority of matched blocks, as found during the initial search, are located in the upper right of the diamond sample plates, thus, the blue point in Figure 3c serves as center. The three red points of the upper right are added, and new diamond search sample plates can be obtained. The small diamond sample plate (red point) is used as the convergence step of the final termination condition, when the center point of the diamond sample plate searched by the block matching algorithm contains the majority of matching blocks, as shown in Figure 3d. In other words, the most matching block in the image can be obtained.

In this paper, mean square error is used for operation of the block matching algorithm, as shown in Equation (1):

(1) $$\text{MSE}\left(i,j,m,n\right)=\frac{1}{N^{2}}\sum_{x=0}^{N-1}\sum_{y=0}^{N-1}{\left(f_{t}\left(m+x,n+y\right)-f_{t-1}\left(m+x+i,n+y+j\right)\right)}^{2}$$

where *f_t_* is the original frame, *f*_*t*__-1_ is the reference frame, *N* is block size, (*x*, *y*) is the pixel within the block, (*m*, *n*) is the starting coordinates of the block, and (*i*, *j*) is the moving coordinates within the search range. In convergence, after calculation of mean square error, Equation (2) is used for further comparison to obtain the minimum mean square error, and the motion vector can be obtained:

(2) $${\left(i,j\right)}_{m,n}=\arg\underset{i,j\in R}{\min}\text{MSE}\left(i,j,m,n\right)$$

where (*i*, *j*)*_m,n_* is the motion vector in block (*m*, *n*), the minimum mean square error of (*i*, *j*), and *R* is the search range.

### 3.2. Skin Color Detection and Intersect Operations

According to the research results of [28], black/yellow/white skin color areas have no great difference in *YCbCr* with concentration features. Thus, *YCbCr* is used as the skin color detection space, and RGB in Equation (3) is converted into a *YCbCr* matrix:

(3) $$\left[\begin{array}{c} Y\\ Cb\\ Cr \end{array}\right]=\left[\begin{array}{c} 0\\ 128\\ 128 \end{array}\right]+\left[\begin{array}{ccc} 0.299 & 0.587 & 0.114\\ -0.169 & -0.331 & 0.5\\ 0.5 & -0.419 & -0.081 \end{array}\right]\;\left[\begin{array}{c} R\\ G\\ B \end{array}\right]$$

Thus, the skin color area can be obtained through Equation (4):

(4) $$\text{Skin}\left(x,y\right)=\left\{\begin{array}{ll} 1, & if\left(\left(60\leq Y\leq 250\right)\cap \left(90\leq Cb\leq 140\right)\cap \left(130\leq Cr\leq 170\right)\right)\\ 0, & \text{otherwise} \end{array}\right\}$$

Intersect operations can combine motion estimation with skin color detection to further obtain dynamic skin color images. In motion estimation, skin color detection can be used to filter non-skin color dynamic areas, such as body motion and dynamic backgrounds. In terms of skin color detection, motion estimation can be used to filter static objects of similar skin colors in the background, and obtain dynamic skin color areas (including dynamic hand gestures). After motion estimation and skin color detection, dynamic skin color images can be obtained through AND operations, as shown in Equation (5):

(5) DSCI(x,y)=MI(x,y)∩SCI(x,y)

DSCI denotes a dynamic skin color image. *MI* and *SCI* are motion images and skin color images, respectively.

In human-machine interaction, commands are sent to computers by hand gestures. Thus, momentum in the hand gesture area is greater than the natural body sway. In projection of dynamic skin color images, filters can be used to filter non-hand gesture areas.

High frequency noise composed of strong peak signals can be removed while maintaining sharpness of edge. The pixel values in the mask are sequenced to determine the intermediate value, and the gray-scale value of the middle pixel in the mask is replaced by the intermediate value. While waiting for output, the location of pixel *h*(*i*,*j*) is taken as the center, an *n* × *n* mask is designated, and all original pixels of the *n* × *n* mask are removed. The set of brightness values is S. The brightness center value Median of the *n*^2^ pixels is found by sequencing, where Median is defined as: (a) Median ϵ S and (b) Median has higher brightness than the half elements of S, (c) Median has lower brightness than the half elements of S, and 9 original pixels are removed from the 3 × 3 mask, with positions and brightness as follows:

$$\begin{array}{c} \begin{array}{cc} \left\{\text{zk}\right\} & =\{10,20,20,20,100,20,20,25,15\}\\ & =\{10,15,20,20,20,20,20,25,100\} \end{array}\\ \text{Median}\{\text{zk}\}=20 \end{array}$$

The intermediate value is “20” after sequencing, which is used as the value of the central pixel *h*(*i*, *j*), which only removes the original isolated high brightness noise “100”, while the brightness values of the peripheral pixels remain approximately unchanged. Median is conducted before open and close. Open and close render the image smooth, while open only processes isolated bright spots, and close only processes isolated dark spots.

Next, the dynamic hand gesture area can be detected through horizontal and vertical projection of the binary dynamic skin color images, and such a projection can filter non-dynamic hand gesture areas. After determining the global maximum peak value, the minimum values can be further determined on both sides of the peak, which are used as intervals of the peak value. Thus, the coordinates within the peak values between horizontal and vertical projections of images are the hand gesture area.

After the dynamic hand gesture area is obtained, features of the detected hand gesture area can be extracted. Robust dynamic hand features can be obtained through feature estimation of dynamic hand gestures, and thus, further increase the recognition rate of dynamic gestures.

### 3.3. Dynamic Hand Gesture Extraction and Feature Analysis

**Figure 4.**
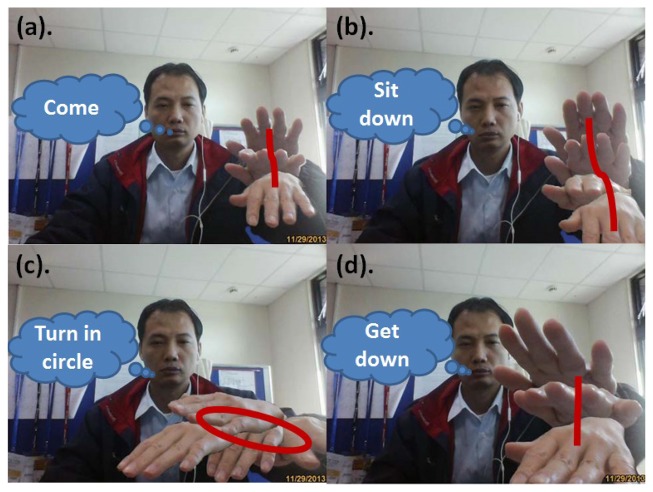
Hand gesture and speech training images.

A complete dynamic hand gesture is a series of image sequence sets. For example, the dynamic hand gesture for “come” is a set of image sequences including waving the arms and palms as shown in Figure 4a. Another example is page turning by animated hands, in which the dynamic hand gestures contain a time correlation. Thus, the movement of a dynamic hand gesture, through continuous images of s computer, is calculated and quantized into different directions (angle) in order to describe hand gesture behavior.

The dynamic hand gesture is extracted as follows: *H_t_* denotes the dynamic hand gesture area detected at time *t*, as shown in Equation (6), where *H_t_*(*x*) and *H_t_*(*y*) are the coordinates of horizontal and vertical pixels in the hand gesture area, respectively:

(6) $$H_{t}=\left(H_{t}\left(x\right),H_{t}\left(y\right)\right)$$

Next, Equation (7) is used to calculate the center of gravity *G_t_* in the hand gesture area at time *t*. The center of gravity is the center point of the hand gesture area, and *x_t_* and *y_t_* are the horizontal and vertical coordinates of center points in the hand gesture area, respectively:

(7) $$G_{t}\left(x_{t},y_{t}\right)=\left(\frac{\sum H_{t}\left(x\right)}{\text{number of x}},\frac{\sum H_{t}\left(y\right)}{\text{number of y}}\right)$$

After the center of gravity in the hand gesture area is obtained, the differential value between vertical and horizontal coordinates in the hand gesture area of current image *t* and the last image *t* – 1 can be calculated through Equation (8), and is expressed by *X_t_* and *Y_t_*. The vector consisting of a differential value is the motion track in the hand gesture area:

(8) $$\left(X_{t},Y_{t}\right)=\left(x_{t}-x_{t-1},y_{t}-y_{t-1}\right)$$

**Figure 5.**
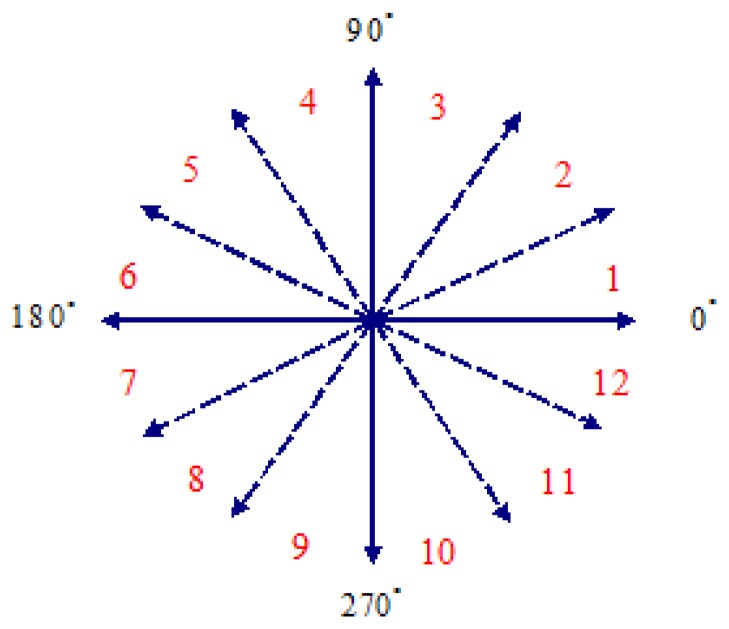
Diagram of angle interval quantization.

Finally, according to the dynamic hand gesture motion track, direction (angle) *θ_t_* is calculated through Equation (9), as shown in Figure 5, where every 30 degrees is regarded as an interval, which is quantized into 12 interval codes and serve as basis of dynamic hand gesture recognition.

(9) $$\theta_{t}=\arctan\left(\frac{Y_{t}}{X_{t}}\right)=\arctan\left(\frac{y_{t}-y_{t-1}}{x_{t}-x_{t-1}}\right)$$

In the detection of dynamic hand gestures, dynamic hand gestures are blurred in a series of image sequence sets, due to hand gesture motions and camera shooting speed. Binary dynamic skin color images in the detection of the dynamic hand gesture area are horizontally and vertically projected, and the hand gesture area has variations (such as inconsistent hand gesture size and position). As a result, extraction of track features is not stable. Moreover, dynamic hand gesture detection technology cannot absolutely detect hand gesture areas. Thus, incorrect dynamic hand gesture detection may cause variations in track feature extraction.

Based on the above feature analysis, in order to reduce instability of motion track due to variations in hand gesture detection areas, a Kalman filter is used to consider the correlation between adjacent hand gesture areas, which can correct motion tracks and obtain robust dynamic hand gesture features to improve the hand gesture recognition rate.

### 3.4. Using Kalman Filter on Time to Estimate Motion Track

A Kalman filter [29,30] contains two phases: estimation and measurement: estimation Equation (10) and measurement Equation (11):

(10) v(k)=Φ(k−1)v(k−1)+Γ(k)w(k)

(11) z(k)=H(k)v(k)+e(k)

The Kalman filter estimation technology is measured through noise, and future recursive program operation is made to correctly estimate system state. In video sequencing, motion of the adjacent area at the time is often highly correlated or consistent. In order to solve the problems of the above features analysis, the correlation of dynamic hand gestures on time and the Kalman filter are used for motion track estimation, thus improving the recognition rate of motion track features.

It is assumed the motion track is treated randomly, and the two components are independent. Thus, the module of the components can be defined, where motion track information is used in the one-dimension auto regressive module, and is the track motion of the center of gravity from the previous frame at the time. One-dimensional regression modules (12) and (13) of the motion track are defined:

(12) $$v_{x}\left(m,n,i\right)=\sum_{p\in S^{⊕}}a_{p}v_{x}\left(m,n,i-p\right)+w_{x}\left(m,n,i\right)$$

(13) $$v_{y}\left(m,n,i\right)=\sum_{p\in S^{⊕}}a_{p}v_{y}\left(m,n,i-p\right)+w_{y}\left(m,n,i\right)$$

where *a_p_* is the module coefficient, which may be a time-based variation or non-variation. In order to simplify the calculation, it is assumed to be a non-variation of time. This study selects the proximal highly correlated adjacent center of gravity for the horizontal and vertical components of the motion track. Thus, Equations (12) and (13) are simplified into Equations (14) and (15):

(14) $$v_{x}\left(m,n,i\right)=a_{1}v_{x}\left(m,n,i-1\right)+w_{x}\left(m,n,i\right)$$

(15) $$v_{y}\left(m,n,i\right)=a_{1}v_{y}\left(m,n,i-1\right)+w_{y}\left(m,n,i\right)$$

In addition, Equations (14) and (15) represent the state space matrix in the execution of Kalman filter regression. Thus, the state space is expressed by the following:

Estimation Equation:
  (16) v(m,n,i)=Φv(m,n,i−1)+Γw(m,n,i)
  where *v*(*m*,*n*,*i*) is the state vector of position (*m*,*n*,*i*), Φ and Γ are corresponding matrixes, and Φ = *a*_1_ = 1, Γ = 1. Thus, Equation (16) can be rewritten as Equation (17):
  (17) $$v\left(m,n,i\right)=a_{1}v\left(m,n,i-1\right)+w\left(m,n,i\right)$$
Measurement Equation:
  (18) z(m,n,i)=Hv(m,n,i)+e(m,n,i)
  where *H* = 1.

The state equation is the quantity equation in the filter. Thus, the Kalman filter calculation is very simple. In a given state space, the general procedure for the Kalman filter is described, as follows:

(1) Estimation
  State estimation:
  (19) $${\hat{v}}^{-}\left(m,n,i\right)=\Phi {\hat{v}}^{+}\left(m,n,i-1\right)$$
  Estimation of co-variance matrix:
  (20) $$P^{-}\left(m,n,i\right)=\Phi P^{+}\left(m,n,i-1\right)\Phi^{T}+\Gamma Q\left(m,n,i\right)\Gamma^{T}$$
(2) Update
  State update:
  (21) $${\hat{v}}^{+}\left(m,n,i\right)={\hat{v}}^{-}\left(m,n,i\right)+K\left(m,n,i\right)\left[z\left(m,n,i\right)-H{\hat{v}}^{-}\left(m,n,i\right)\right]$$
  Update-error co-variance:
  (22) $$P^{+}\left(m,n,i\right)=\left[I-K\left(m,n,i\right)H\right]P^{-}\left(m,n,i\right)$$
  Kalman gain matrix:
  (23) $$K\left(m,n,i\right)=P^{-}\left(m,n,i\right)H^{T}{\left[HP^{-}\left(m,n,i\right)H^{T}+R\left(m,n,i\right)\right]}^{-1}$$

In a Kalman filter algorithm, Kalman gain depends on *q*(*m*,*n*,*i*) and *r*(*m*,*n*,*i*). In state update, *q*(*m*,*n*,*i*) and *r*(*m*,*n*,*i*) can be used to decide the number of predicted values or measured values of reference states. According to the analysis results, the greatest distances are caused by errors or instability in the detection of the dynamic hand gesture area in the current frame. Thus, one exponential function concept is used to approximate variance *q*(*m*,*n*,*i*), as shown in Equation (24). The detection of the current hand gesture area has error or instability when the difference of the hand gesture features (motion track) is great. Thus, this study intends to use more estimated values of state (motion track in the previous hand gesture area on the time) in order to correct the current measured motion track; otherwise, more measured values are considered, as shown in Equation (25):

(24) $$q\left(m,n,i\right)=\exp\left(c\left(\sqrt{{\left(z_{x}-{\hat{v}}_{x}^{-}\right)}^{2}+{\left(z_{y}-{\hat{v}}_{y}^{-}\right)}^{2}}\right)\right)$$

(25) r(m,m,i)=1−q(m,m,i)

where *c* is a normalized parameter, and *q*(*m*,*n*,*i*) is normalized from 0 to 1. Here, *c* is set to −0.1.

### 3.5. Detection of Repetitive Gesture Area and Autocorrelation Function Operation

In human-machine interaction, hand gestures are repeated when users give the same command or intention, and times of repetition are not consistent in each time. For example, the hand gesture “come” is a set of waving a hand up and down, and even two or three times as shown in Figure 4a. Thus, while the times of repetition are not consistent, the gesture is repeated. In view of this, repetitive hand gestures may cause variations in feature extraction, and affect dynamic gesture recognition accuracy.

The traditional gesture recognition system extracts hand gesture features in a series of input image sequences, but fails to consider the impact of repetitive hand gesture segments on recognition. This study detects the repeatability of hand gesture segments, with gesture signals as input through Autocorrelation Function operations and estimation, and extracts one gesture signal from repetitive hand gesture segments. It is then quantized into 12 directional codes as input vectors for training and recognition of dynamic hand gestures in order to reduce variations in feature extraction.

**Figure 6.**
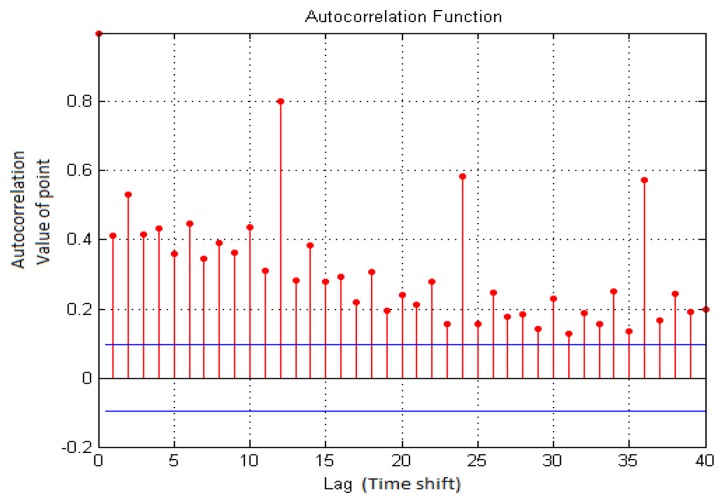
Detection of repetitive hand gesture segment using autocorrelation function.

Based on the above analysis results, in the detection of repetitive speech or gesture segments, this study uses the autocorrelation function to detect repetitive hand gestures or speech signal segments in continuous images or speech sequences, as shown in Equation (26):

(26) $$r_{xx}(k)=\frac{1}{M}\sum_{n=1}^{M}x(n)\times x(n+k)$$

where *k* is time displacement (the frame on the time axis or sound frame displacement); *r_xx_*(*k*) is the autocorrelation function of time displacement *k*; *x*(*n*) is the input signal at time n (hand gesture motion track or Mel-frequency cepstral coefficients); *M* is the total length of the input signal. *M* represents the number of repeated sections to be selected. As shown in Figure 6, the detection result of the dynamic hand gesture for “come” in the repetitive gesture segments is described. Lag represents the X-axis, which represents time shift, and the Y-axis represents the modified correlativity value after autocorrelation. The repeatability of 12, 24 and 36 is seen. Finally, the autocorrelation degree 0.8 point Value of Lag12 is used as the eigenvector model. According to the analysis result, there is one repetitive gesture segment between wave crests on the curve. When making the hand gesture for “come”, the users may wave a hand five times. In order to reduce feature detection variation caused by repetitive gesture segments, features of the first repetitive hand gesture segment are extracted after detecting the repetitive gesture segment. The features are then quantized into 12 directional codes, which are used as the feature basis for future training and recognition of dynamic hand gestures.

## 4. Speech Recognition Technology

**Figure 7.**
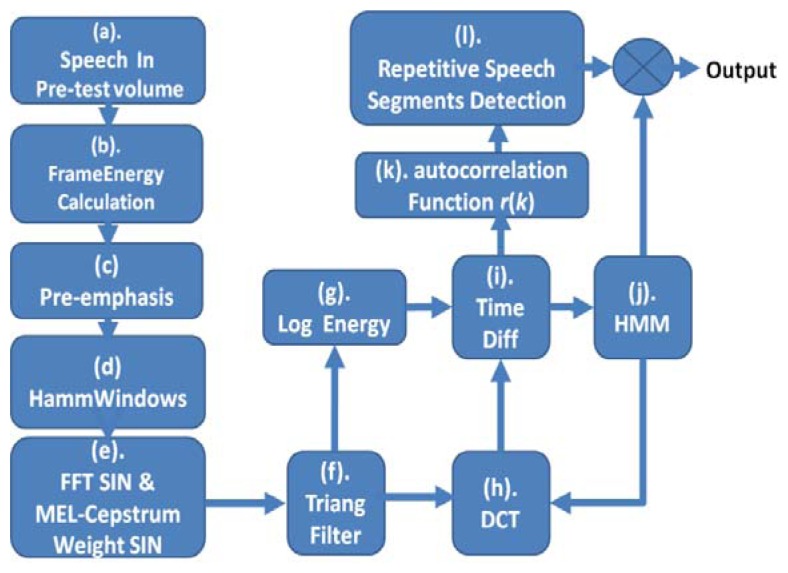
Feature extraction flow chart of Mel-frequency cepstral coefficients.

Speech recognition has the same repetitive segment as gesture recognition. In order to avoid feature extraction features due to inconsistent times of repetition of speech commands, detection of repetitive segments is required for hand gestures. First, Mel-frequency cepstral coefficients (MFCC) are determined from audio signals, and used as input. In Figure 7, the autocorrelation function operation (Equation (26)) is made to detect repetitive speech segments, and extract the MFCC of the single repetitive speech segment.

**Figure 8.**
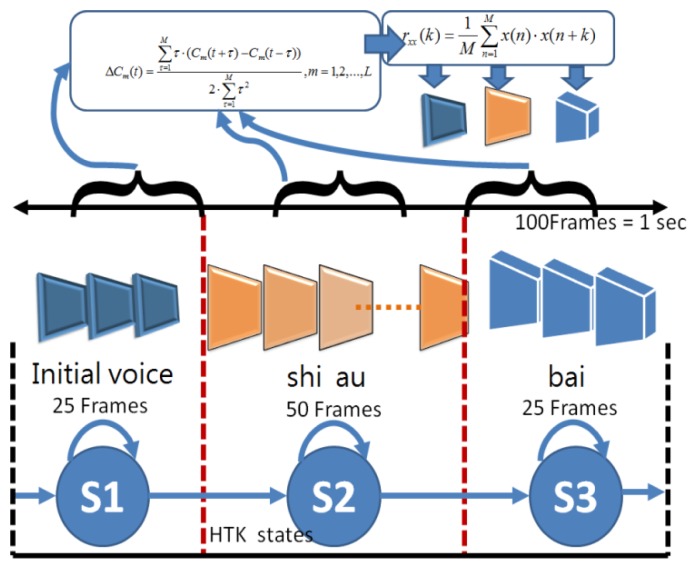
Speech recognition block.

Autocorrelation and HMM operation steps include: (a) the speech signal is obtained from the environment, and the speech and image recognition system functions are started; (b) and (c) the speech input signal is compensated by pre-emphasis; (d) framing process, in order to observe the feature of sound signal and take a number of sampling points to collect observations, and the signal is multiplied by a window function for statistical calculation; this paper uses a Hamming Window; (e) and (f) use FFT sine and cosine functions to represent the composite wave of the waves of different cycles for each original signal, where the composition of the original signal can be obtained by FFT in order to observe the energy distribution in the spectrum, obtain the MEL-Cepstrum Weight value, and adjust the coefficient of the triangular filter, which is exported to the triangular filter of (f) to calculate the Bank of Spectral energy in different triangular filter frequency bands, and determine the harmonic series represented by repeated sections in the frequency domain and its correlation function, which is then exported to (g) to calculate the energy of the nonlinear logarithm. In (h) the N-order characteristic coefficients are determined by DCT; (i) the time difference and the energy value of the previous and next frames are used to calculate the distance. Finally, the energy vector value of each frame is exported to (k) and (j), where (k) is a frame energy vector value determined by autocorrelation, and (j) HMM is used to train the energy vector value of each frame in each state till the state model converges. The results can serve as the input for the speech command recognition model, in order to establish human behaviors. The speech recognition block process is shown in Figure 8, which according to Figure 7 steps include (d),(e),(f),(g),(h),(i),(k). The calculation procedure is described as follows:

Speech Input:

waveFile = ‘SpeechWave.wav’;
[y, fs, nbits] = wavread(waveFile).

**Figure 9.**
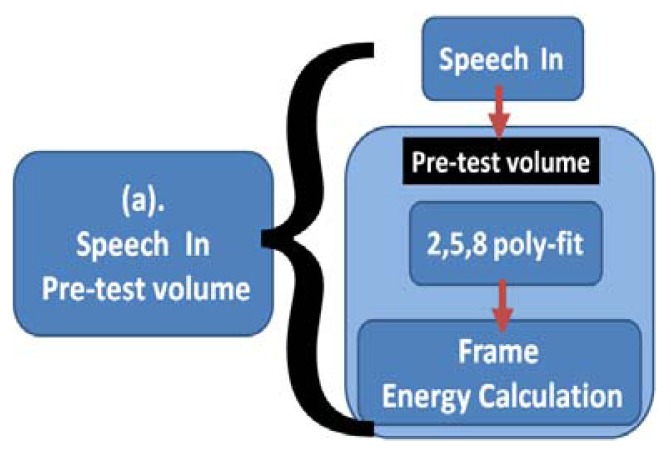
Speech In pre-test volume.

In Figure 9, different corpora real time recording forecast volumes are imported according to Speech In. This paper considers the corpus recording volume; however, not all corpus systems have a consistent recording volume to avoid overflow of the buffer. This paper modifies the representative computing equation for one frame energy, as shown in Equation (27). Adds *Ev*, takes No. 2, 5, and 8 frames, uses poliy-fit to determine the average curve of the frame, and calculates the average volume; where there are 10 levels, from level 1 to level 10. *Ev* will automatically adjust the range according to the 10 levels. When the volume of a recorder or corpora is high, we can select factors that can be dynamically adjusted by *Ev* = *e*^6^ or higher, as shown in Equation (28).

Now in order to reconstruct an exponential function, we have to exponentiate the fitting line *y* = *ax* + *b*, where *fit*(1) = *a* and *fit*(2) = *b*, it can be rewritten as *e^ax^*^+^*^b^* = *e^b^* + *e^ax^*

Matlab coding:

*x* = *e*-data(:,1);
*y* = *e*-data(:,2);
fit = polyfit(*x*, log(*y*),1);

So we can plot the data with the exponential fit as:

semilogy(*x*, *y*, ‘o’, *x*, *exp*(*fit*(2)).*exp(*fit*(1)*x))
  (27) $$\text{energy}=\sum_{n=1}^{\text{frameSize}}x{\left(n\right)}^{2}$$
  (28) $$\text{energy}=\sum_{n=1}^{\text{frameSize}}\left(\overset{x{\left(n\right)}^{2}}{\;}/\underset{E_{v}}{\;}\right),\;E_{v}=403=e^{6}$$

If one short time interval is a frame, when the sound signal feature is observed, a number of points are sampled from the signal for collective observation. When a frame after finishing, as shown in Equation (29):

(29) $$\tilde{M}[n]=\hat{x}[n]\times w[n]$$

In this paper we adopt Hamming Windows to design amplification constant. The traditional approach is shown in Equation (30):

(30) $$w(n,\alpha )=(1-\alpha )-\alpha \cos\left(\frac{2\pi n}{N-1}\right),0\leq n\leq N-1$$

where *w*(*n*,*α*) is windows function:

(31) $$w[n]=\{\begin{array}{cc} x(n)\times \text{hammTable}\;(n), & 1\leq n\leq \text{frameSize}\\ 0, & \text{otherwise} \end{array}$$

In order to avoid buffer data overflow, and for convenient identification of data dispersion degree (Overflow Data with both Discrete Discrimination, *ODDD*), an amplification coefficient *ODDDv* is designed:

(32) $$\begin{array}{cc} w[n]=x(n)\times \text{hammTable} & \left(n\right)/\text{ODDDv}\\ \text{ODDDv}=2^{\text{alp}}, & 1\leq n\leq \text{frameSize} \end{array}$$

**Figure 10.**
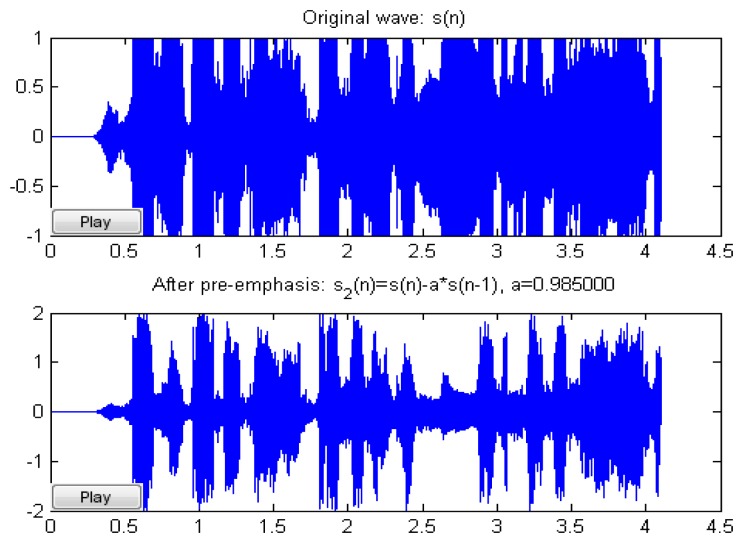
Original wave compare with Humaning Windows.

**Figure 11.**
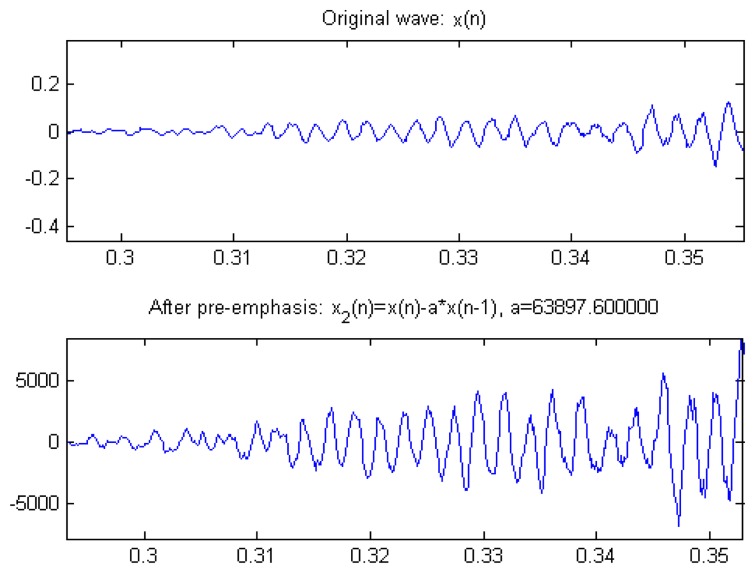
The typical pre-emphasis magnification.

Speech signal Sinput (*n*) is imported *x_out_*(*n*) = *x_input_*(*n*) – *α* × *x*(*n*–1) in order to compensate for elimination of the suppressed high audio frequency of human pronunciation. Generally, the human labial cut-off point parameter is set as 0.95∼0.985, *n* is the time factor, and the scale is automatically adjusted for improvement. Figure 10 shows the Original wave compare with Humaning Windows. Figure 11 shows the typical pre-emphasis magnification, parameter value *α* is 0∼1. After automatic adjustment of scale, *α* is 2^15^, and magnification is 63,897.6, where *x* [*n*] represents the sound signal, *n* is the time factor, *α* = 0.975, and the common range is 0.95∼0.958.

The sound is acoustically different from the spectrum, and resolution at low frequency is higher than that at high frequency. Therefore, pre-emphasis shall be conducted, where the transfer function of pre-emphasis is expressed, as shown in Equation (33):

(33) $$M^{'}(n)=\left(x(n)\times 2^{\text{alp}}-63898\times x(n-1)\right)/2^{\text{alp}},2\leq n\leq \text{frameSize}$$

The mask spreading function is due to two reasons, one is the cepstrum, which may result in hundreds of multidimensional features, the other is that people cannot distinguish between similar frequencies. As a phonetic feature, it can be reduced to MFCC by this phenomenon. When the repeated feature is determined, the vector model of one frame is selected from the repeated frames as the feature model. This study aims to determine repeated sections in a continuous section.

**Figure 12.**
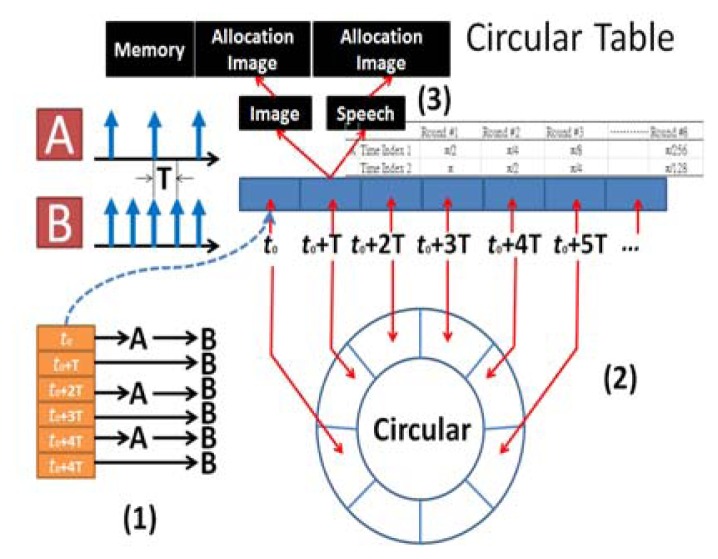
SIN Circular Table.

In weight cepstrum (WC), the typical practice is the Taylor expansion. This paper creates a SIN Circular Table of 12 integral WC, and the SIN operation of FFT has 18 integer values as shown in Figure 12(A: Image frames, B: Speech frames). In Figure 12(1), the temporary address of each dynamic gesture and speech SIN integer parameter includes timing, Index, *etc.* In Figure 12(2), for the space defined by these addresses, circular polling is adopted to save memory space, and improve query speed and accuracy. In Figure 12(3), the merged image and speech vectorial character models are stored in memory according to the respective time stamps.

**Figure 13.**
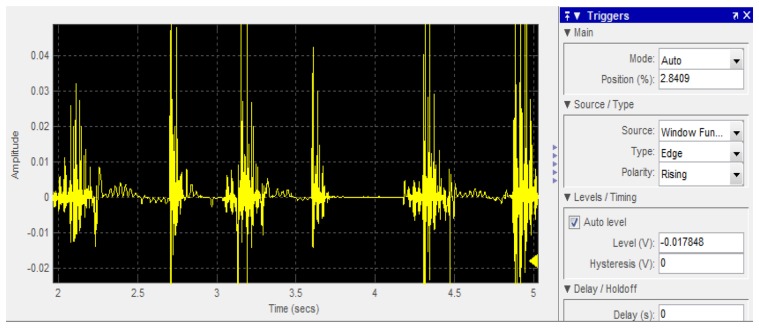
Multiplied by Hamming Windows time domain distribution.

**Figure 14.**
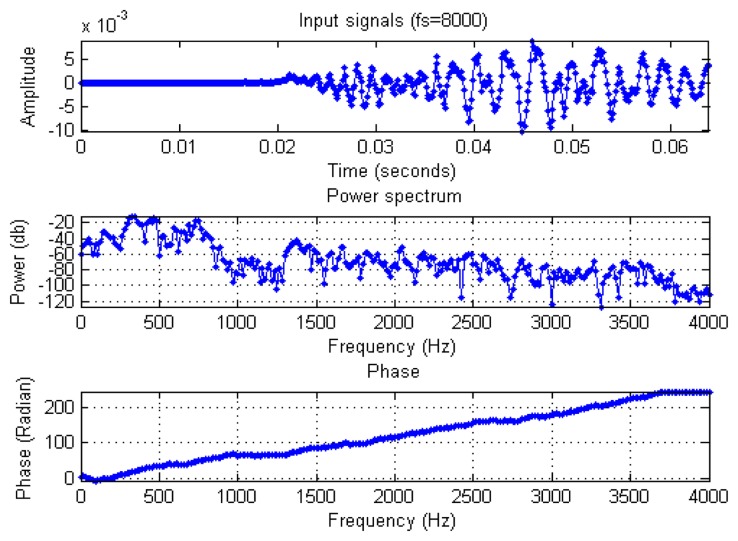
Frequency domain energy distribution of FFT.

The 18 integer mathematic values of SIN converted by FFT are marked, the SIN Circular Table of WC is calculated, the SIN operation of FFT has 18 integer values, and the computing values of this stage have a SIN Circular Table of 30 elements as shown in Table 1. According processes block in Figure 7, we used Hamming window to process signal and output it within the FFT calculation and transfer of SIN function, as shown in Figures 13 and 14:

(34) $$F(k)=\sum_{n=0}^{N-1}\tilde{x}(n)E_{N}^{kn},0\leq k\leq N-1,\;\text{where}\;E_{N}=e^{-jk2\pi /N}$$

where *F(k)* is *x̃*[*n*] Fourier transform, *k* represents the *k*-th Frequency Bin.

Define a mel-scale:

$\text{mel}-\text{scale}=2595\times \log_{10}\left(\frac{f}{700}+1\right)=2595\times \log_{10}\left(\frac{1000}{700}+1\right)=999.885..$
$\text{mel}-\text{scale}=2595\times \log_{10}\left(\frac{f}{700}+1\right)=2595\times \log_{10}\left(\frac{500}{700}+1\right)=607.446‥$
$\text{mel}-\text{scale}=1125\times \ln\left(\frac{f}{700}+1\right)=1125\times \ln\left(\frac{500}{700}+1\right)=606.371..$

**Figure 15.**
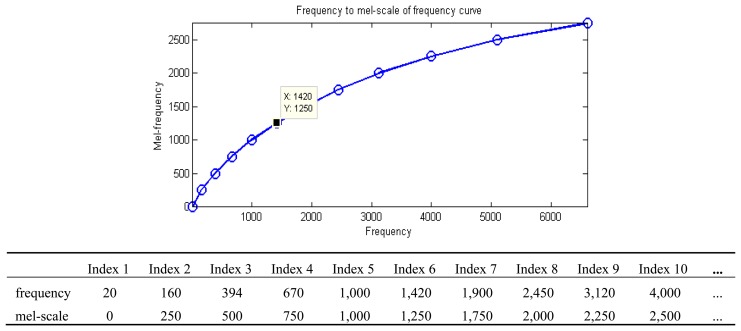
Frequency to mel-scale of frequency curve.

The mel-scale is calculated isometrically, and the value of the corresponding frequency of x-axis is calculated and stored in the corresponding Circular Table. The integer value of the look-up table is searched by Binary Search, *i.e.*, the value in the corresponding Time index and the previously integralized logarithmic value, in order to avoid directly use of the rooting function of a floating-point number. The corresponding rooting table can increase the computing speed, reduce the memory space occupied by the rooting table, and maintain a certain amount of accuracy. The signal in each frame is processed by MFCC in order to obtain the spectral energy value parameter of the signal in the frame as shown in Figure 15.

**Figure 16.**
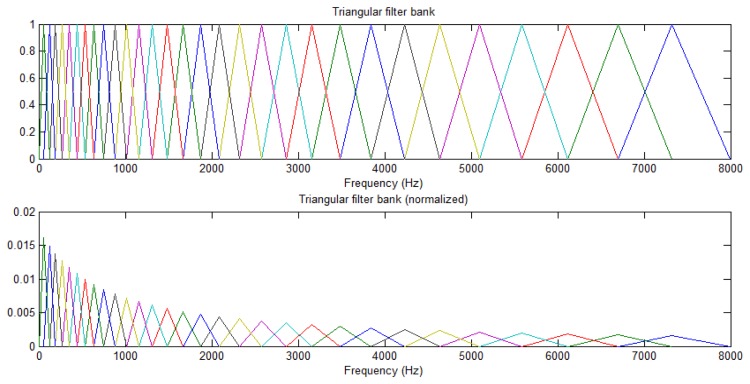
Triangle of band-pass filter in Spectrum.

Using the Mel-filter to signal processing each frame, and its can be calculated spectral energy value of the parameters:

(35) $$\begin{array}{c} \begin{array}{ccc} M[m]=\overset{N-1}{\underset{k=0}{\text{∑}}}{\left|X_{a}[k]\right|}^{2}H_{m}[k] & ,0<m\leq L, & L:\text{number of channel} \end{array}\\ \begin{array}{l} {\left|X_{a}[k]\right|}^{2}=\{\begin{array}{ll} x_{r}[k]=x_{r}[k]/\text{byte} & \text{real number},4\;\text{bytes}\\ x_{t}[k]=x_{t}[k]/\text{byte} & \text{imaginary unit},4\;\text{bytes}\\ \sqrt{x_{r}{\left[k\right]}^{2}+x_{t}{\left[k\right]}^{2}}\times \text{byte} & \text{elements},\text{total}=2^{8}=256 \end{array}\\ \begin{array}{l} H_{m}[k]=\{\begin{array}{ll} 0 & \;k\leq f[m-1]\\ \frac{\left(k-f[m-1]\right)}{\left(f[m]-f[m-1]\right)} & \;f[m-1]<k\leq f[m]\\ \frac{\left(f[m+1]-k\right)}{\left(f[m+1]-f[m]\right)} & \;f[m]\leq k<f[m+1]\\ 0 & \;k>f[m+1] \end{array} \end{array} \end{array} \end{array}$$

where *M* [*m*] is the m-th triangular of band-pass filter of spectrum energy of value, the L is a triangular of band-pass filter, *H*_m_[*k*] is the *m*-th triangle band-pass filter function and |*X_a_*[*k*]|^2^ is a spectral of energy values, the triangular of band-pass filter in Spectrum domain as shown in Figure 16. While the feature is determined by similar cepstrum (e.g., Diff-cepstrum), the action state columns approach to synchronization in this paper, which consider the speed, and whether there is repeated semantic and recoverable features, such as noise interference.

Discrete Cosine Transform Process of step in Figure 7h block. MFCC of FFT signal is the frequency domain within that we are used a discrete cosine transform (DCT) method transform to signal of frames in time-domain analysis, when its gets spectrum energy from the filter group, and whichever of the values into the discrete cosine transform is obtained the characteristics of N-order factor, where *N* is a factor of numbers setting to 30. Discrete cosine transform formula is as follows:

(36) $$c_{m}=c\sum_{j=1}^{N}m_{j}\cos\left(\frac{\pi i}{N}(j-0.5)\right),c=\sqrt{\frac{2}{N}}=\sqrt{\frac{2}{30}}=0.27735$$

where *i* = *number of MFCC*, *N* = *number of band*—*pass filter*. *M_j_* is a *Log* (*M*[*m*]), the *m*-th triangular of band-pass filter of spectrum energy of log value. Performing discrete cosine transformation, that its can adjust the weights *w*(*i*) the value of the energy for the filter bank to turning its, show the formula as follows:

(37) $$c_{m}=c\overset{N}{\underset{j=1}{\text{∑}}}w(i)m_{j}\cos\left(\frac{\pi i}{N}(j-0.5)\right)$$

where *w*(*i*) is the *i*-th triangular band-pass filter weights. Details of calculating the features based on MFCCs. The first order regression coefficients are computed by the following regression Equation:

(38) di=∑m=1Mm(Cm(m+i)−C(m−i)m)2∑τ=1Mτ2,m=1,2,…,L

where *d_i_* is the delta coefficient at frame *i* computed in the corresponding basic coefficients *C_m_*_+_*_i_* to *C_m_*_−_*_i_*:

(39) ΔMm(t)=∑τ=−MMτCm(t+τ)∑τ=−MMτ2=∑τ=1Mτ(Cm(t+τ)−C(t−τ)m)2∑τ=1Mτ2,m=1,2,…,L

Autocorrelation and crosscorrelation are common concepts for calculating signal analysis, and represent correlativity between the values of two time series, as obtained at two different time points in the same time series, respectively. Namely, the cross correlation function describes the correlativity between the values of stochastic signals *r_x_*_1_(*k*) and *r_y_*_2_(*k*), as obtained at two different time points *t*_1_ and *t*_2_. The autocorrelation function describes the correlativity between the values of stochastic signal *r_xx_*(*k*), as obtained at two different time points, *k*_1_ and *k*_2_ as shown in Equation (26). The cross correlation function gives a judgment index of correlation between two signals in the frequency domain connecting the cross spectrum and autospectrum of signals between two measuring points, and determines how much of the output signal is derived from the input signal, which is very effective at correcting measurement errors resulted from accessing a noise source.

The correlation coefficient is merely a ratio, is neither an equivalent unit measure, nor a correlated percentage, and is generally two places of decimals. The sign of a correlation coefficient only represents the correlated direction, while the absolute value represents the degree of correlation. As it is not an equivalent unit measure, the correlation coefficient of 0.7 is not twice 0.35, but the correlativity between two columns of variables with a correlation coefficient of 0.7, which is higher than the correlativity between two columns of variables with correlation coefficient of 0.35. In addition, the increased correlation coefficient from 0.70 to 0.80 cannot be regarded as identical to an increased correlation coefficient from 0.30 to 0.40. The values of correlation coefficient are expressed, as shown in Table 2.

## 5. Multimodal Model Integration

The traditional human behavior recognition method only considers single media information, such as image or speech. The variations in feature extraction may lower the total recognition rate if only speech or an image is considered in human behavior recognition, meaning human behaviors cannot be correctly deduced. In addition, linear combinations of the image recognition model and speech recognition model can improve single model recognition. However, it has difficulty in deducing the weight (importance) of image and speech models for human behavior recognition in complicated natural environments, thus, the correction effect is limited.

The fusion of multi-sensor information is based on mathematical derivation of statistics:

(40) $${\text{P}(\text{T}}_{\text{k}}{/\text{S}}_{1}{,\text{S}}_{2}{,\text{S}}_{3},..{\text{S}}_{\text{n}}{)=\text{P}(\text{S}}_{1}{/\text{T}}_{\text{k}})\times {\text{P}(\text{S}}_{2}{/\text{T}}_{\text{k}})\times \ldots {\text{P}(\text{S}}_{\text{n}}{/\text{T}}_{\text{k}})/\sum {\text{P}(\text{S}}_{1}{/\text{T}}_{\text{i}})\times {\text{P}(\text{S}}_{2}{/\text{T}}_{\text{i}})\ldots {\text{P}(\text{S}}_{\text{n}}{/\text{T}}_{\text{i}})$$

where *P*(*T_k_*/*S_1_*,*S_2_*,*S_3_*……,*S_n_*) represents the probability of attaining the goal, and *T_k_* represents the multi-sensor environment. The basic thought of the model probability of the two forecast examples is described as follows:

(1) Suppose image sensor tracker (T_1_) computes a feature vector for track #1, denoted as T_1_: T_1_ = {5.0,10.0, 75.0, 60.0, 2.0, 150.0, 75.0, 20.0}, and suppose the audio sensor for the microphone tracker (T_2_) outputs a feature vector for track #2, denoted as T_2_: T_2_ = {10.0, 40.0, 85.0, 65.0, 2.0, 140.0, 65.0, 85.0}. The correlation coefficient between the two feature vectors is equal to 0.87. Therefore, the result of the fusion action is that track #1 and track #2 are two distinct tracks.
(2) Suppose the image sensor tracker produces a feature vector for track #1, denoted as T_3_: T_3_ = {30.0,20.0, 60.0, 70.0, 2.0,100.0, 60.0, 30.0}, and suppose the Audio Sensor for the microphone tracker defines a feature vector for track #2, denoted as T_4_: T_4_ = {30.0, 20.0, 60.0, 70.0, 2.0, 100.0, 60.0, 30.0}. The correlation coefficient between the two feature vectors is equal to 1.0. Therefore, the result of the fusion action is that track #3 and track #4 most likely characterize the same target.

**Figure 17.**
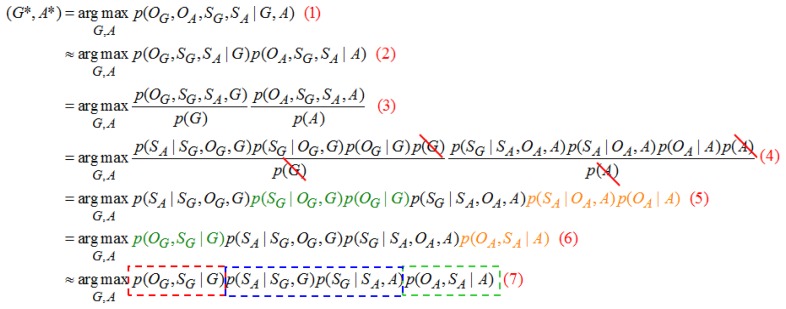
Gesture and speech recognition model adopted Bayesian theorem.

During recognition of human actions, in addition to speech command recognition and hand gesture recognition models, the corresponding relationship between state sequences of the hand gesture model and speech model is further considered in order to increase the accuracy of human action recognition. The mathematical expressions are defined as follows:

When the hand gesture recognition and speech recognition models are considered, it is expected to consider the potential coincidence relation in state sequence between models, thus, in Figure 17(1) approximates as shown in Figure 17(2). In Figure 17(2) can be resolved into Figure 17(3) by the Bayes theorem. As the coincidence relation between gesture and speech models states that sequences are unrelated to the observation probability, it is neglected to obtain Figure 17(4). The present input gesture and speech observation data are recognized with given gestures and speech model parameters, where a group of the most matching (*G,A*) model parameters are determined by input gesture and speech observation data, and the action type of (*G,A*) is the final recognition result. *T* is enumerating every possible state sequence of length *T*; there will be N_T_ possible combinations of state sequence where *N* is the total number of states. Suppose there is one state sequence S_G_ and it is set of {*G_1_*, *G_2_*,…*G_T_*}:

(41) $$\begin{array}{l} \begin{array}{ll} \left(G*,A*\right) & =\underset{G,A}{\arg\max}p(O_{G},O_{A},S_{G},S_{A}|G,A)\\ & \approx \underset{G,A}{\arg\max}p(O_{G},S_{G},S_{A}|G)p(O_{A},S_{G},S_{A}|A)\\ & =\underset{G,A}{\arg\max}p(O_{G},S_{G}|G)p(S_{A}|S_{G},O_{G},G)p(S_{G}|S_{A},O_{A},A)p(O_{A},S_{A}|A)\\ & \approx \underset{G,A}{\arg\max}p(O_{G},S_{G}|G)p(S_{A}|S_{G},G)p(S_{G}|S_{A},A)p(O_{A},S_{A}|A) \end{array} \end{array}$$

(*G*,*A*) is the hand gesture and speech model of the same action: *p*(*O_G_*,*S_G_*∣*G*) and *p*(*O_A_*,*S_A_*∣*A*) are the hand gesture and speech command recognition models, respectively; *p*(*S_A_*∣*S_G_*,*G*) shows the correspondence probability of speech state sequence *S_A_* in hand gesture model *G* after a certain hand gesture state sequence *S_G_* is given; *p*(*S_G_*∣*S_A_*,*A*) is the correspondence probability of the hand gesture sequence *S_G_* in speech command model *A* after a certain speech state sequence *S_A_* is given. Regarding recognition, the biggest action category (G*,A*) of the posterior probability *p*(*O_G_*,*S_G_*∣*G*) *p*(*S_A_*∣*S_G_*,*G*) *p*(*S_G_*∣*S_A_*,*A*) *p*(*O_A_*,*S_A_*∣*A*) is the human behavior of the last recognition.

**Figure 18.**
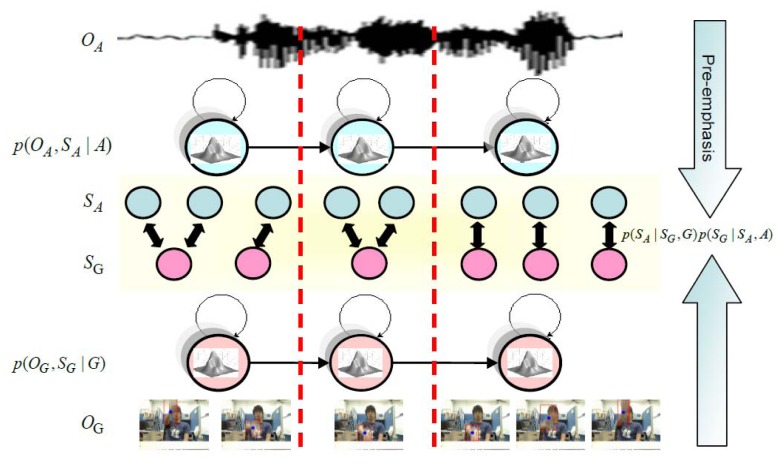
Multimodal model merging.

The proposed multimodal model integration is as shown in Figure 18. For the input sequence (hand gesture *O_G_* and *O_A_*), where detection of repetitive hand gestures and speech segments is completed, hand gestures and speech can be recognized through the hidden Markov model. The corresponding relationship between state sequences of hand gestures and speech model state sequences is further considered. In the hand gesture model, the correspondence between state sequences of the hand gesture recognition model and the speech recognition model should be considered, besides itself. Likewise, in the speech recognition model, the correspondence between state sequences of the speech recognition model and hand gesture recognition model should also be considered.

The proposed multimodal model merging method can be divided into two parts for description. In training phase, feature extraction is made for collected parallel data (input of hand gestures and speech commands). For hand gestures, a multimode Kalman filter is used to extract features for re-estimation and autocorrelation functions in order to detect repetitive hand gesture segments. For speech, the repetitive speech segment is detected by using autocorrelation functions.

**Figure 19.**
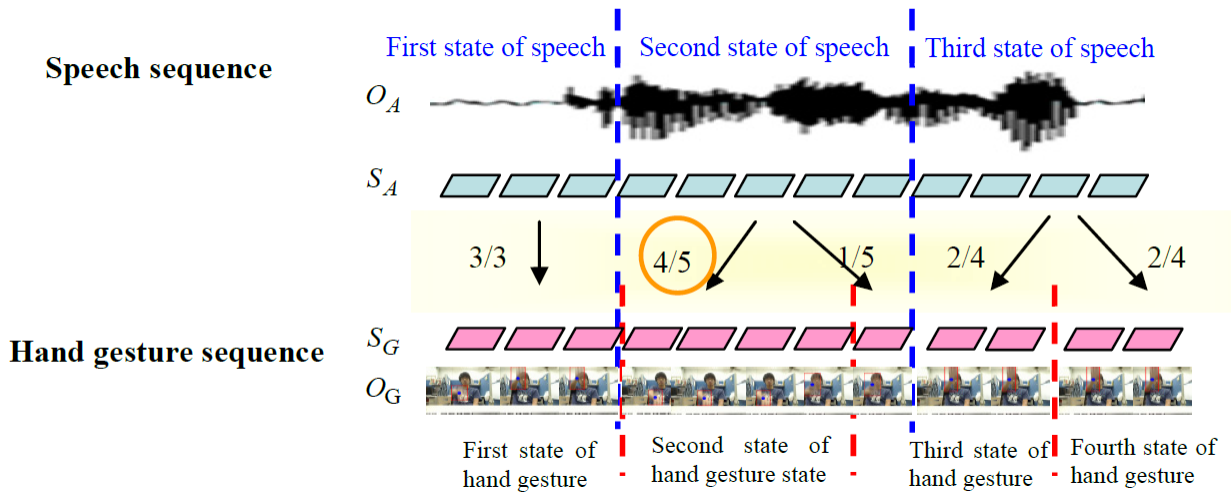
Correspondence between state sequences of speech and hand gesture.

Next, the hand gesture and speech Hidden Markov models are separately trained to obtain the optimal state sequence and the correspondence. For example, the blue dotted lines in Figure 19 represent a speech sequence boundary, while the red dotted lines represent a hand gesture state sequence boundary. The speech model has three states, and the hand gesture model has four states. The second state of speech corresponds to the fifth hand gesture image, the fourth state corresponds to the second state of the hand gesture, and the first state corresponds to the third state of the hand gesture. From this, the probability of the second state of speech corresponding to second state of hand gesture is four fifths. Through the correspondence method, the correspondence between state sequences of hand gesture and speech can be obtained through the correspondence of the collected training data.

**Figure 20.**
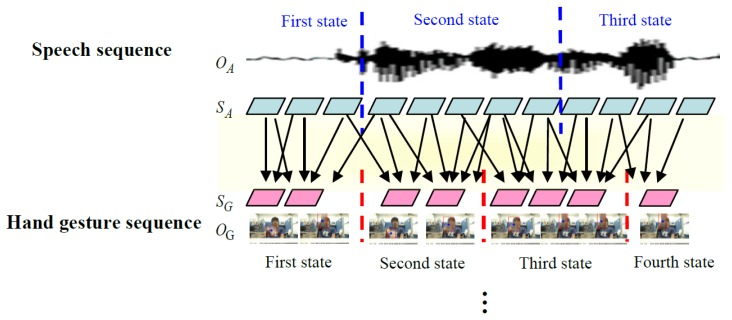
Correspondence between state sequences of optimal speech and hand gesture.

In the testing phase, for the input state sequences of hand gestures and speech, where detection of repetitive segments is completed, the recognition result is obtained through the model established before training. Moreover, the recognition result should be corrected through correspondence between the state sequences of hand gestures and speech. Figure 20 illustrates correspondence between the optimal hand gesture and speech state sequence in the testing phase. For recognition results of the hand gesture and speech models, the n paths are separately listed (the n paths are reserved after completion of Viterbi). For each path, correspondence between state sequences in training is used to calculate correspondence between the optimal hand gesture and speech in this path. Finally, an optimal path can be found, and maximum probability can be obtained by Equation (41). The human behavior of maximum probability is the final recognition result.

## 6. Simulation and Results

This study used a PC configured with Pentium 4 3.0 G and 1 G for all tests. The programs were developed using Borland C++ Builder 6.0, and performed by a Logitech “Logitech QuickCam™ Sphere” (with inbuilt microphone).

### 6.1. Experiment System Flow Chart

**Figure 21.**
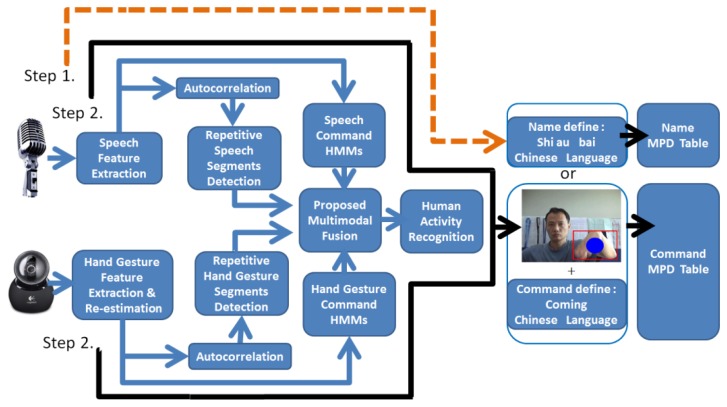
Human behavior recognition flow chart.

Figure 21 shows the human behavior recognition system flow chart. First, we extract the input image and speech sequences from a webcam and microphone. In hand gesture, we extract the hand gesture area by the dynamic hand gesture detection technique. Furthermore, the Kalman filter and autocorrelation function can be used to reduce variations in feature extraction. In speech, after extraction of the Mel-scale frequency, the Cepstral coefficients autocorrelation function can be used to reduce the variations of feature extraction in repetitive speech segments. Finally, human behavior is recognized through multi-modal model merging.

In a special application case, human behavior interacts with a machine in a situation based on the interaction between an electronic pet dog and human behavior. This system defines 32 actions and one EPD Name, where one user (keeper) can raise multiple pets (one File is opened for independent EPD). The EPD Game system executes keeper authority, the first UI is entered, and the EPD randomly migrates in a 3D scene. When the volume energy heard through the microphone is greater than the threshold, Step 1 (dotted line) is entered, and the recording action is initiated. This step has no hand gesture recognition, and the training name is repeated more than five times. Each training and previous model value iterative constructs model training. After the second recognition, if the system recognition is correct, the EPD wags its tail. A question mark occurs if recognition is wrong. Step 2 is the path of the training “command-action” model (solid line), which switches to another command edit menu for 32 “command-action” training, where speech and gesture synchronization actions are trained each time, with the procedure of speech similar to the aforesaid.

**Figure 22.**
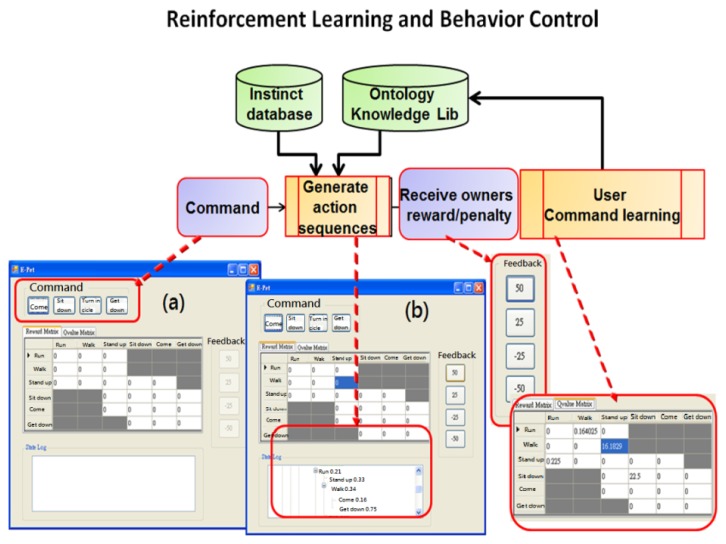
Reinforcement learning and behavior control.

Example: naming, each sampling time is 5 s, repeat speech sampling, repeat three times. Figure 22 shows the behavior sequence uses a Markov state probability decision-making form for MPD training, where in Figure 22a is the preliminary training state of one command, and in Figure 22b action sequence is randomly generated. In each training round, the EPD generates a behavior sequence according to the action table in MPD, and guesses a relevant action, where errors will be punished through a reward and punishment system, correct or incorrect will reward-penalty {50, 25, −25, −50} value.

**Figure 23.**
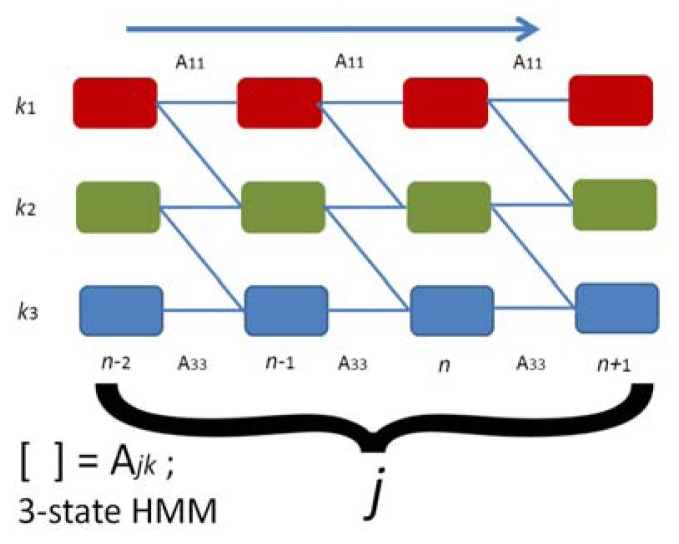
It is a 3-state HMM array by A_jk._

The complete parameter set *λ* of the HMM parameters for hand gestures represent vector *π* and two matrices A and *ϕ*, where HMM parameter set *λ* is for training the parameters of the state model. The probability of the vector model is evaluated by observing the maximum likelihood performance of observable output symbol sequence *S*. The probability of the maximum likelihood of the state sequence is represented by (*S*∣*λ*). It is a 3-state HMM array by A_jk_ as shown in Figure 23.

Determine optimal state sequence:

The HMM parameter set λ is given to observe output observable symbol sequence *G* in order to determine an optimal {*G_1_*; *G_2_*;…*G_T_*} state sequence of *S*_G_. The Viterbi algorithm is applied to determine the single best state sequence *S*_G_ = {*G_1_*; *G_2_*;…*G_T_*} state sequence. We give the observable symbol sequence *S* = {*s_1_*,…,*s_T_*} and the HMM parameter set l in order to maximize *P*(*S*_G_∣*S*, λ), it can be written as:

(42) $$P\left(S_{G}|S,\lambda \right)=\frac{P\left(S_{G},S\begin{array}{c} |\lambda \end{array}\right)}{P\left(S\begin{array}{c} |\lambda \end{array}\right)}$$

(43) $$w\left(z_{n}\right)=\begin{array}{c} \max\\ z_{1},z_{2}‥,z_{n-1} \end{array}P\left(s_{1},\ldots s_{n},z_{1},\ldots ,z_{n}\right)$$

**Figure 24.**
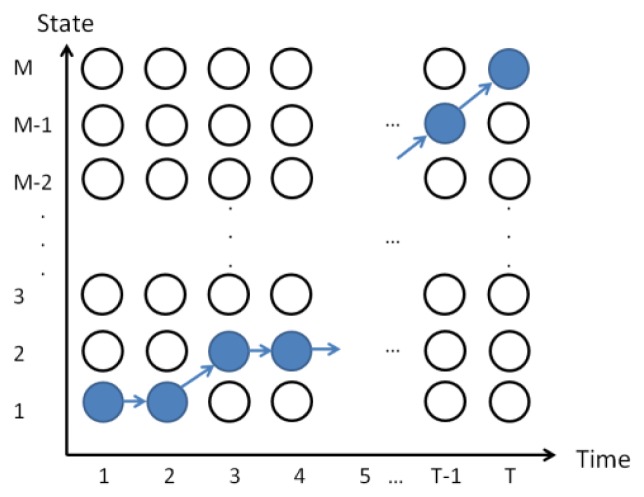
Viterbi algorithm path.

**Figure 25.**
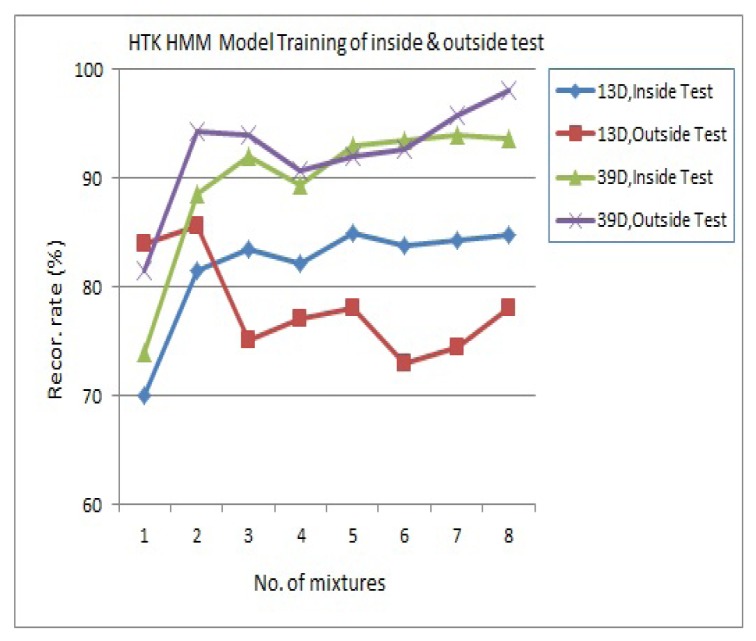
HMM model training.

The best state transfer process is selected according to the given model and observed sample sequence, and the most probable state of the picture frame/voice at time t can be found, as shown in Figure 24. Finally, the feature model is determined by parameter estimation using the baum-welch method. The process of HMM parameters for speech recognition is the same as above, *S_A_*={*A_1_*,*A_2_*…*A_T_*}. SA is the speech using the Viterbi algorithm to find the single best state sequence. This paper uses HTK for HMM testing of 13D-dimensional inside test and outside test, where the recognition rate is apparently increased in 39D dimensions, as shown in Figure 25.

### 6.2. Experimental Settings and Evaluation

**Figure 26.**
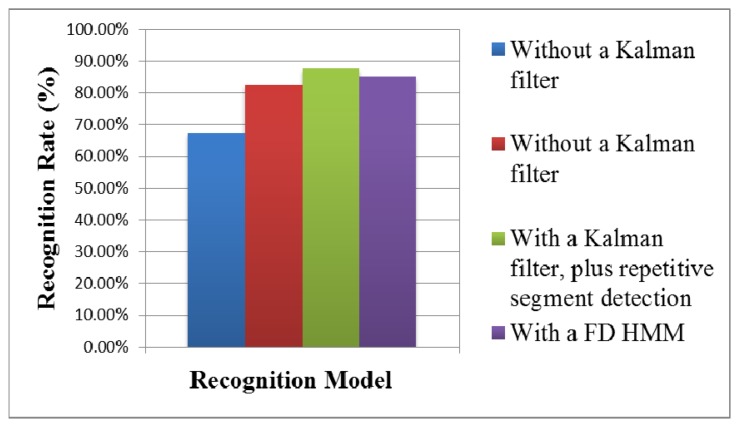
The recognition results of dynamic hand gestures.

**Figure 27.**
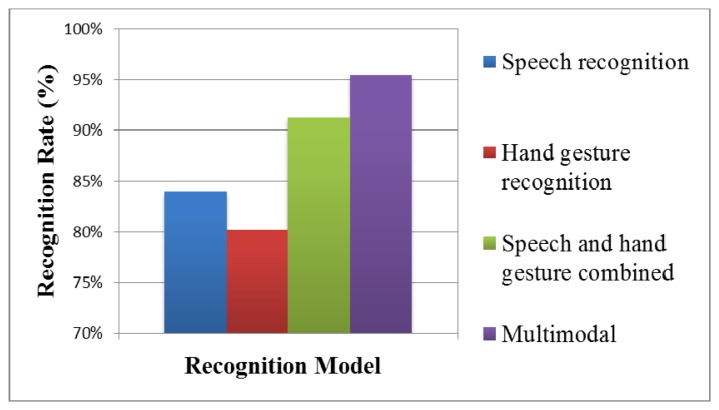
The correct recognition rate of the recognition models.

The experimental results can be divided into two parts, as per the system flow chart:

(1) Hand gesture recognition results
  In dynamic hand gesture detection, two students made four different hand gestures: come, sit down, turn in circle, and get down as shown in Figure 4. Each student repeated each hand gesture five times. In each time, 60 images were extracted, and the frame format was SIF. Each hand gesture had 600 images for evaluation, as shown in Table 3. The total number of tested images (including four actions) was 2,400, among which dynamic hand gesture images from correct recognition was 2,225, and dynamic hand gesture images from wrong recognition was 175, with an average detection rate of hand gestures of 92.7%.
  The recognition results without a Kalman filter, with a Kalman filter, with a Kalman filter plus repetitive segment detection, and with a FD HMM [26], were compared, as shown in Tables 4, 5, 6 and 7 and Figure 26. The two students made four hand gestures, including come, sit down, turn in circle, and get down. Each person made one gesture five times. In the recognition results of 40 dynamic hand gestures, the correct recognition of dynamic hand gestures without a Kalman filter was 27 times, the wrong recognition without a Kalman filter was 13 times, with an average recognition rate of 67.5%. The correct recognition of dynamic hand gesture with a Kalman filter was 33 times, and wrong recognition with a Kalman filter was seven times, with an average recognition rate of 82.5%. The correct recognition of dynamic hand gesture with a FD HMM was 34 times, and wrong recognition with a FD HMM was 6 times, with an average recognition rate of 85%. With a Kalman filter, plus repetitive segment detection, the correct recognition of the dynamic hand gestures was 35 times, and wrong recognition was 5 times, with an average recognition rate of 87.5%. The recognition result is better than that without Kalman filter or with Kalman filter.
(2) Results of speech recognition and multi-modal model
  Regarding human behavior action recognition, the Hidden Markov Model Toolkit [31] was used for recognition. The hand gesture recognition model is a hidden Markov model, with six states from left to right (including start to end). The speech recognition model is a hidden Markov model, with five states from left to right (including start to end).
  In training the recognition model, and tested 10 males and five females in the experiment room performed four different actions: come, sit down, turn in circle, and get down as shown in Figure 4. Each action was repeated 20 times, which served as trained data. In testing, each person repeated each hand gesture 10 times and each action 20 times. The recognition results in the speech recognition model, hand gesture recognition model, traditional linear combination of speech recognition model, with a hand gesture recognition model, were compared with that of the proposed multimodal model, as shown in Figure 27. The correct recognition rate of the speech recognition model was 84%; the correct recognition rate of the hand gesture recognition model was 80.25%; the correct recognition rate of the traditional speech and hand gesture combined recognition model was 91.25%; the correct recognition rate of the proposed multimodal model was 95.5%. Experimental results verified that the multimodal gesture-speech model provided superior accuracy when compared to the single modal versions. The test result shows that the recognition is reduced by about 2.5%. It is preliminarily found that there is influence when two conditions are tenable. First, the subjects have similar tones, e.g., similar pronunciations of Coming and Getting. Second, the tracks and lengths of the gestures of Coming and Sitting are similar; therefore, the recognition rate decreases when the two conditions are tenable. However, it is stabilized at 95.5% when the number of trainings is increased by 20.

## 7. Conclusions

This study proposed a dynamic hand gesture detection and recognition system. The preliminary experimental results confirmed that the proposed method can effectively detect dynamic hand gesture areas in complicated natural environments. The proposed hand gesture feature re-estimation and detection technology of repetitive hand gesture segments can effectively improve the recognition rate of dynamic hand gestures. In the future work, we will combine Ontology and Q-table to implement 32 action command using speech spotting and gesture spotting.

In this study, a speech recognition model, with correspondence between state sequences of hand gestures and speech models were further considered. The experimental results proved that the proposed multi-modal model can effectively improve human action recognition. The recognition rate of a traditional HTK running MFCC is about 80%–85%, where image recognition is implemented by the above recommended method (vector model trained by direction parameter and quantified by movement track). The single test recognition rate is about 80%. The combined recognition rate of speech and hand gestures is increased to 91%, and the recognition rate is increased to 95% by the multi-model integration of Bayes.

## Figures and Tables

**Table 1.** SIN Circular Table value.

	**Round #1**	**Round #2**	**Round #3**	**…………**	**Round #8**
Time Index 1	π/2	π/4	π/8		π/256
Time Index 2	Π	π/2	π/4		π/128

		**Time Index 1**	**Time Index 2**
A	Round #1	π/512	π/256

**Table 2.** The values of correlation coefficient.

**Correlation Coefficient**	**Correlativity Interval**
0.00–±0.30	Slight correlation
±0.30–±0.50	Actual correlation
±0.50–±0.80	Significant correlation
±0.80–±1.00	High correlation

**Table 3.** Accuracy of dynamic hand gesture.

**Hand Gesture**	**Come**	**Sit Down**	**Turn in Circle**	**Get Down**

**Diction Result**				
Number of tested images	600	600	600	600
Correct detection	554	517	570	584
Wrong detection	46	83	30	16
Accuracy	92.3%	86.2%	95.0%	97.3%

**Table 4.** Confusion matrix for recognition results of dynamic hand gestures without a Kalman filter.

**Hand Gesture**	**Come**	**Sit Down**	**Turn in Circle**	**Get Down**

**Input Model**				
Come	9	1	0	0
Sit down	1	9	0	0
Turn in circle	2	1	3	4
Get down	1	1	2	6

**Table 5.** Confusion matrix for recognition results of dynamic hand gestures with a Kalman filter.

**Hand Gesture**	**Come**	**Sit Down**	**Turn in Circle**	**Get Down**

**Input Model**				
Come	9	1	0	0
Sit down	1	9	0	0
Turn in circle	1	1	7	1
Get down	1	0	1	8

**Table 6.** Confusion matrix for recognition results of dynamic hand gestures with a Kalman filter, plus repetitive segment detection.

**Hand Gesture**	**Come**	**Sit Down**	**Turn in Circle**	**Get Down**

**Input Model**				
Come	9	1	0	0
Sit down	1	9	0	0
Turn in circle	0	1	9	0
Get down	1	0	1	8

**Table 7.** Confusion matrix for recognition results of dynamic hand gestures with a FD HMM.

**Hand Gesture**	**Come**	**Sit Down**	**Turn in Circle**	**Get Down**

**Input Model**				
Come	8	1	0	1
Sit down	2	8	0	0
Turn in circle	0	0	9	1
Get down	0	1	0	9

## References

[1] Calinon S., Billard A. Incremental Learning of Gestures by Imitation in a Humanoid Robot.

[2] Calinon S., Billard A. A Framework Integrating Statistical and Social Cues to Teach a Humanoid Robot New Skills.

[3] Varkonyi-Koczy A.R., Tusor B. (2011). Human-computer interaction for smart environment applications using fuzzy hand posture and gesture models. IEEE Trans. Instrum. Meas..

[4] Manresa C., Varona J., Mas R., Perales F.J. (2000). Real-time hand tracking and gesture recognition for human-computer interaction. Electron. Lett. Comput. Vis. Image Anal..

[5] Dardas N.H., Georganas N.D. (2011). Real-time hand gesture detection and recognition using bag-of-features and support vector machine techniques. IEEE Trans. Instrum. Meas..

[6] Ren Z., Yuan J., Meng J., Zhang Z. (2013). Robust part-based hand gesture recognition using kinect sensor. IEEE Trans. Multimed..

[7] Chen Q., Georganas N.D., Petriu E.M. (2008). Hand gesture recognition using haar-like features and a stochastic context-free grammar. IEEE Trans. Instrum. Meas..

[8] Frolova D., Stern H., Berman S. (2013). Most probable longest common subsequence for recognition of gesture character input. IEEE Trans. Cybern..

[9] Zhang X., Chen X., Li Y., Lantz V., Wang K., Yang J. (2011). A framework for hand gesture recognition based on accelerometer and EMG sensors. IEEE Trans. Syst. Man Cybern. Part A Syst. Hum..

[10] Stern H., Edan Y. (2005). Cluster labeling and parameter estimation for the automated setup of a hand-gesture recognition system. IEEE Trans. Syst. Man Cybern. Part A Syst. Hum..

[11] Xu R., Zhou S., Li W.J. (2012). MEMS accelerometer based nonspecific-user hand gesture recognition. IEEE Sens. J..

[12] Tang H.-K., Feng Z.-Q. Hand's Skin Detection Based on Ellipse Clustering.

[13] Zhang M.-J., Wang W.-Q., Zheng Q.-F., Gao W. Skin-Color Detection Based on Adaptive Thresholds.

[14] Cai X., Jiang L., Hao X.-W., Meng X.-X. A New Region Gaussian Background Model for Video Surveillance.

[15] Wang S.B. (2008). Video Completion Based on Effective Spatial and Temporal Inpainting Techniques. M.Sc. Thesis.

[16] Yoon H.-S., Chi S.-Y. Visual Processing of Rock, Scissors, Paper Game for Human Robot Interaction.

[17] Han J., Award G.M., Sutherland A., Wu H. Automatic Skin Segmentation for Gesture Recognition Combining Region and Support Vector Machine Active Learning.

[18] Hayakawa H., Shibata T. Spatiotemporal Projection of Motion Field Sequence for Generating Feature Vectors in Gesture Perception.

[19] Lin W., Sun M.-T., Poovandran R., Zhang Z. Human Activity Recognition for Video Surveillance.

[20] Ghimire D., Lee J. (2013). Geometric feature-based facial expression recognition in image sequences using multi-class adaboost and support vector machines. Sensors.

[21] Tang X., Liu Y., Lv C., Sun D. (2012). Hand motion classification using a multi-channel surface electromyography sensor. Sensors.

[22] Henry P., Krainin M., Herbst E., Ren X., Fox D. RGB-D Mapping: Using Depth Cameras for Dense 3D Modeling of Indoor Environments.

[23] Henry P., Krainin M., Herbst E., Ren X., Fox D. (2012). RGB-D Mapping: Using kinect-style depth cameras for dense 3D modeling of indoor environments. Int. J. Robot. Res..

[24] Yang X., Yuan J., Thalmann D. Human-Virtual Human Interaction by Upper Body Gesture Understanding.

[25] Shen X., Hua G., Williams L., Wu Y. (2012). Dynamic hand gesture recognition: An exemplar-based approach from motion divergence fields. Image Vis. Comput..

[26] Chen F.S., Fu C.M., Huang C.L. (2003). Hand gesture recognition using a real-time tracking method and hidden Markov models. Image Vis. Comput..

[27] Tham J.Y., Ranganath S., Ranganath M., Kassim A.A. (1998). A Novel unrestricted center-biased diamond search algorithm for block motion estimation. IEEE Trans. Circuits Syst. Video Technol..

[28] Chai D., Bouzerdoum A. A Bayesian Approach to Skin Color Classification in YCbCr Color Space.

[29] Liu C.-S., Lin J.-C., Yang N.-C., Kuo C.-M. Motion Vector Re-estimation for Trans-coding Using Kalman Filter.

[30] Kuo C.-M., Chung S.-C., Shih P.-Y. (2006). Kalman filtering based rate-constrained motion estimation for very low bit rate video coding. IEEE Trans. Circuits Syst. Video Technol..

[31] Young S., Evermann G., Gales M., Hain T., Kershaw D., Liu X., Moore G., Odell J., Ollason D., Povey D. (2009). The HTK Book (for HTK version 3.4); Engineering Department.

